# To Study the Clinical, Biochemical and Radiological Features of Acute Pancreatitis in HIV and AIDS

**DOI:** 10.4021/jocmr1040w

**Published:** 2013-01-11

**Authors:** Shahzad Raza, Naueen A. Chaudhry, Jordan D. Brown, Sina Aghaie, Damoun Rezai, Areej Khan, Paul De Leon Tan, Barbara J. Berger

**Affiliations:** a Department of Internal Medcine, Brookdale University Hospital and Medical Center, New York, NY, USA; b Division of Infectious Diseases, Brookdale University Hospital and Medical Center, New York, NY, USA

**Keywords:** Pancreatitis, HIV, AIDS, Highly Active Antiretroviral Therapy (HAART), Hepatitic C virus, Hepatitis B virus

## Abstract

**Background:**

Pancreatitis complicating HIV infection, even in the Highly Active Antiretroviral Therapy (HAART) era, remains a management challenge. We felt there is a need to discern patterns in the biochemical markers, radiological studies, co-infections, length of stay (LOS) in patients with HIV or AIDS AND pancreatitis.

**Methods:**

This is a retrospective study conducted from June, 2008 to August, 2010 on patients admitted with acute pancreatitis to our hospital. We extracted and compared the following parameters: biochemical markers, HBV markers (surface antigen, core antibody and surface antibody), HCV antibody, radiological studies, and length of stay (LOS). The Balthazar Grade score was used to assess radiological severity of disease. We stratified the cohort into comparison subsets according to CD4 count.

**Results:**

Ninety-four admissions met the criteria for HIV or AIDS AND pancreatitis; 67 unique patients comprised the cohort. Median age was 48 years (range, 23 to 60 years). Thirty seven (55%) were male, 30 (45%), female. Two third (n = 51) (76%) were African American. Known risk factors included a history of pancreatitis, 17 (25%); cholecystitis, 13 (19%); alcohol abuse, 25 (37%); Intravenous drug abuse, 18 (27%). Only 36 (38%) admissions were on HAART regimen. Biochemical features on admission were: WBC, 6,100/mm^3^ (900 - 25,700); amylase, 152 U/L (30 - 1,344); lipase, 702.5 U/L (30 - 5,766), triglyceride, 65 mg/dL (57 - 400); glucose, 94 mg/dL (60 - 1,670); lactate, 2.3 mmol/L (1.09 - 5.49); AST, 61.5 U/L (9 - 1,950); LDH, 762 U/L (394 - 5,500); bicarbonate 19.5 mEq/L (3.3 - 82.7). Interestingly, 62% patients had normal pancreas on CT scan on admission. Of 67 individuals, hepatitis profile was available in 43, 21 (49%) were positive for HCV, 11 (26%) had markers for HBV. Four of 11 patients (36) with CD4 < 50 had evidence of persistent HBV (+core, -surface ab). Patients with CD4 < 200 have a median time for hospital course of 8 days (range 4 - 61 days) compare to 3 days in patients with CD4 > 200. P = 0.03 via t-test comparison. One patient with CD4 < 50 died due to acute pancreatitis.

**Conclusion:**

Pancreatitis remains a major cause of morbidity in HIV-infected individuals. This study has provided detailed features in the HAART therapy era about the clinical, biochemical and radiological features of pancreatitis. Half of our patients were positive for HCV; additionally, 36% with CD4 < 50 had persistent HBV. As opposed to earlier studies, we did not find a female predominance. Patients with CD4 < 200 had a 2.67-fold increase length of stay. Future studies are needed for a closer look on viral cofactors which might precipitate episodes of acute pancreatitis.

## Introduction

Pancreatitis has been a common cause of morbidity and occasional mortality in patients with HIV infection and AIDS [1]. The annual rate of pancreatitis in healthy people is generally low, 17 to 30 cases per 100,000. However, the annual rate of acute pancreatitis in Unites States HIV population is considerably higher, and has even been reported as many as 14 cases per 100 HIV patients in a one year period [2-4]. Patients with AIDS often present with acute pancreatitis (4-22%) and it has also been reported either as a complication of the disease as well as the therapy [4-5]. An important study by the EuroSIDA investigators involving more than 9000 patients, studied from 2001 to 2006, showed that there was a ‘low overall rate of pancreatitis’ (1.27 cases of pancreatitis per 1,000 patient-years) in HIV-1 patients [3-5].

The pathophysiology of pancreatitis in HIV and AIDS may or may not be similar to patients who don’t have HIV. The absence of a consensus on a definition of pancreatitis, inconsistencies in the collection and analysis of clinical and laboratory data, and the lack of long-term follow-up have led to variability in the reporting of the epidemiology of Pancreatitis.

Risk factors associated with pancreatitis in HIV and AIDS patients are several including history of ethanol use, gallstones, biliary disease and hypertriglyceridemia. With advancing disease, opportunistic infections like Cytomegalovirus, Cryptosporidiosis, Mycobacterial disease) and prophylactic therapy (for example, pentamidine) also cause pancreatitis. Now, with the continuously widening spectrum for treatment options Antiretroviral medications (for example, Nucleoside Reverse Transcriptase Inhibitors (NRTIs) particularly Didanosine and Stavudine), Non-nucleoside Reverse Transcriptase Inhibitor (NNRTI’s ), Protease Inhibitors (PI’s) , Corticosteroids, Ketoconazole, Sulphonamides, Metronidazole and Isoniazid can also be risk factors in HIV patients [6, 7].

Since the widespread availability of combination HAART therapy, the study of risk factors of acute pancreatitis in HIV infected patients is due for frequent revisits [7].

The present study is designed to determine primarily the contributing risk factors, biochemical markers, and radiological features of pancreatitis associated with HIV and AIDS. Secondary objectives include the frequency of co-infections in HIV/AIDS pancreatitis and to collect information on length of stay in both groups, HIV and AIDS pancreatitis.

## Material and Methods

### Patients and data collection

The Brookdale University Hospital and Medical Center is a New York State AIDS-Designated Center located in East Brooklyn. We provide longitudinal primary and subspecialty care as well as acute hospital care for patients in the Brownsville/East New York region of Brooklyn, NY (NYC). We retrospectively reviewed the records of all patients discharged with the diagnosis codes for HIV (V08) or AIDS (042) AND pancreatitis (755) from June 2008 to August 2010. According to institutional policy, at the time of admission into the hospital, all patients undergo a detailed baseline assessment with collection of demographic, medical, social, behavioral, and clinical information; and comprehensive panel of blood tests, which include biochemical markers, Hepatitis B virus markers (surface antigen, core antibody and surface antibody), Hepatitis C virus antibody, Hepatitis profile, HIV status with CD4 count. These data were available as routine clinical information and the study collected only information which was available in the course of patient care. Length of stay was calculated from the admission and discharge data of the patient records. Death or discharge information was also collected from the medical record. We used Balthazar Grade scoring for assess severity of acute pancreatitis [8]. Maintenance of the database and use of its contents for analysis of patient outcomes is approved by the Institutional Review Board of the Brookdale University Hospital and Medical Center. Table 1 describes the demographics of our study population.

**Table 1:** Demographics of Study Population

Patient Characteristics	N	%
No of Admissions	94	
No of Patients	67	
Age (Median)	48	(Range 23 - 60)
Gender		
Male	37	55
Female	30	45
Race		
African American	51	76
Hispanic	13	19
Others	3	5
Hepatitis Profile (n = 43)		
Hepatitis B (sAg +)	11	26
Hepatitis C	21	49
Alcohol Abuse (n = 53)	27	51
Patients on HAART therapy	36	38
Prophylaxis for opportunistic infection		
Pentamidine	1	2
Co-Trimoxazole	13	34
Atovaquine	4	10.5
IV Drug abuse (n = 49)	18	37

### Definitions and statistical analysis

For the purpose of our study, acute pancreatitis was defined on the basis of typical history and clinical features with supporting biochemical evidence of pancreatitis (elevated serum amylase or lipase three times the upper limit of normal) [1, 7]. For our analysis, HIV transmission risk factors included injection drug use (IDU), men who had sex with men (MSM), and heterosexual transmission (defined as either heterosexual activity with a partner at high risk for HIV or sex with an HIV-infected individual).

Prior antiretroviral therapy was categorized as none, NRTI’s, NNRT’s, protease inhibitors, and integrase inhibitors in the combination of HAART. HAART was defined as concomitant use of three or more antiretroviral drugs either from two or more classes (NRTIs, non-nucleoside reverse transcriptase inhibitors (NNRTIs), Protease inhibitors, or a fusion inhibitor) or three NRTIs. Currently used antiretroviral agents were those that had been used in the 3 months prior to the admission with the diagnosis of pancreatitis. Death was attributed to this diagnosis if the patient died during the defined hospitalization for pancreatitis and HIV or AIDS.

Descriptive statistics were performed using SPSS version 13 service pack. We compared the characteristics of two groups of patients: one with HIV AND pancreatitis; the other with AIDS AND pancreatitis. This comparison was based on the clinical, biochemical, and radiological features of patients in each of the two groups. A review was also done of hospital LOS between HIV patients with pancreatitis and those without, and also of AIDS patients with and without pancreatitis. Univariate and Multivariate conditional logistic regression analysis was used to assess the risk factors for the initial episode of pancreatitis.

## Results

Between June 2008 and August 2010, Ninety-four episodes of acute pancreatitis were analyzed. Sixty-seven unique patients were identified. Eight patients were admitted twice; four patients were each admitted with three episodes; one patient was admitted with four episodes; and two with five episodes of acute pancreatitis. Among 67 patients, 37 (55%) were male and 30 (45%) were female. African American ethnicity was predominant in our population (n = 51) (76%) and Hispanics were (n = 13) (19%). The median age of our study cohort was 48 years (range, 23 to 60 years). Thirteen patients (19.4%) had a previous history of cholecystectomy prior to the admission.

Based on our pool of data, only 36 patients were receiving HAART therapy. Fifteen of the 36 were taking the combination of tenofovir and emtricitabine (Truvada). We described the number of each NRTIs, NNRTI, protease inhibitors and integrase inhibitors received by our patient population at the time of acute pancreatitis in Table 2.

**Table 2:** Patients Receiving HAART Therapy at the Time of Pancreatitis

HAART Therapy n=36	No of Patients
Nucleoside reverse transcriptase inhibitors (NRTIs)	
Emtricitabine	17
Zidovudine	17
Tenofovir	22
Abacavir	6
Non-nucleoside reverse transcriptase inhibitors (NNRTIs)	
Efavirenz	10
Protease Inhibitors	
Lamivudine	22
Atazanavir	7
Darunavir	3
Ritonavir	17
Lopinavir	5
Nelfinavir	3
Saquinavir	1
Integrase Inhibitor	
Isentress	3

Eighteen patients (37%) had a positive history of intravenous drug use. Forty three patients from among sixty-seven, had a hepatitis profile available. Twenty-one (49%) were positive for HCV antibody, 11(26%) for hepatitis B surface antigen (HBsAg). The CD4 count was assessed at each episode of pancreatitis. Thirty-four (53%) patients had a CD4 count less than 200 cells/mm^3^ at the time of their episode of pancreatitis. Table 3 describes frequency of acute pancreatitis with CD4 count at the time of admission. One patient died during the hospital course. Of all 38 episodes of pancreatitis in AIDS patients, 13 patients were receiving co-trimoxazole, four patients received atovaquone and one patient received pentamidine for *Pneumocystitis jiroveci* prophylaxis.

**Table 3:** Acute Pancreatitis and CD4 Counts

CD4 count (cells/mm^3^)	n = 72	%
≤ 50	23	32
51 - 200	15	21
201 - 350	13	18
350 - 500	12	16.6
> 500	9	12.5

In univariate regression model, we did not find a statistically significant factor which can affect pancreatitis (Female gender (P = 0.4), ethnicity (P = 0.09), HAART therapy (P = 0.9)). However, we found an increase in the incidence of pancreatitis in patients with CD4 count less than 50. This feature was statistically significant, P = 0.03, (CI; 2.2 - 3.80 compared to those with CD4 count > 200. For patients with CD4 count below 50 (n = 11), 36% (n = 4) patients had chronic hepatitis B infection (based on +ve Core Ag and -Cve HBSAb). Patients with AIDS and low CD4 count < 200 also had a LOS of eight days (median range 4 -61 days) compared to three days (2 - 61days), P = 0.03 via t-test comparison. Hence patients with CD4 < 200 had a 2.67-fold increase length of stay.

### Biochemical

Among the cases of pancreatitis, the median amylase level was 152 U/L (range, 30 - 1,344 U/L; normal 30 - 110), and the median lipase level was 702.5 U/L (range 30 - 5,766 u/L; normal 23-300). Triglyceride levels were 65 mg/dl (57 - 400). White blood cell count was 6,100/mm^3^ (range, 900 to 25,700). Table 4 describes biochemical characteristics of acute pancreatitis.

**Table 4:** Biochemical Characteristics of Acute Pancreatitis

Lab Values	Median	Range	Normal Range
Amylase (U/L)	152	30 - 1,344	30 - 110
Lipase (U/L)	702.5	30 - 5,766	23 - 300
Triglycerides (mg/dL)	65	57 - 400	40 - 150
WBC No/mm^3^	6,100	900 - 25,700	4 - 11
Glucose (mg/dL)	94	60 - 1,670	65 - 99
Lactate (mmol/L)	2.3	1.09 - 5.49	0.7 - 2.1
AST (U/L)	61.5	9 - 1,950	17 - 59
Bicarbonate (mEq/L)	19.5	3.3 - 82.7	22 - 28

### Radiology

**Figure 1:**
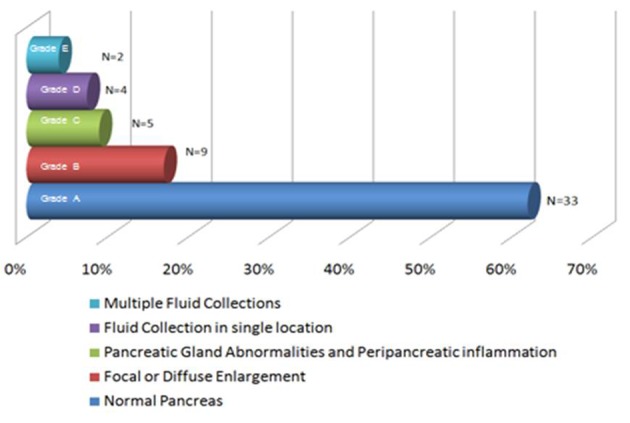
The representation of acute pancreatitis and Balthazar grade scoring system.

**Figure 2:**
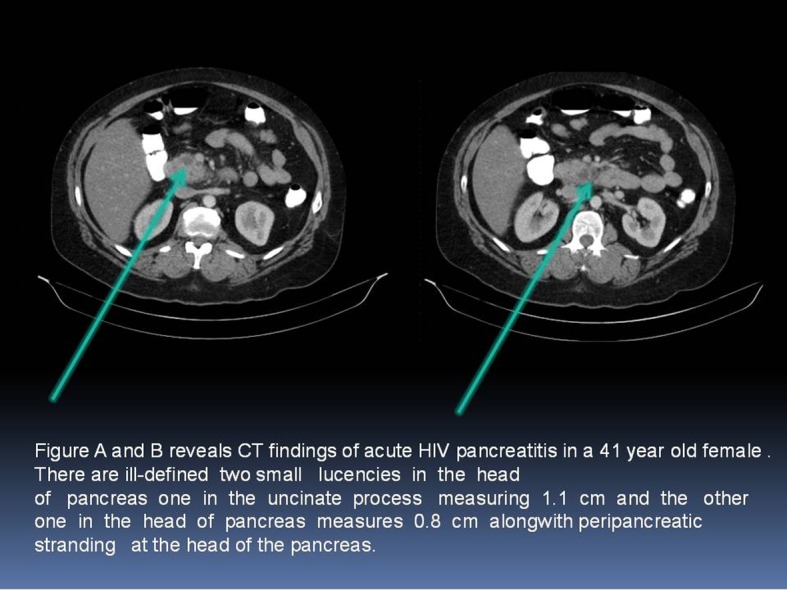
A and B: CT scan of acute pancreatitis in a 41-year-old female showing lucencies in the head of the pancreas and peripancreatic fluid stranding.

CT scan of the abdomen with intravenous contrast was performed in 53 patients within 48 hours of admission. We used Balthazar grade scoring system to define the radiological changes in acute pancreatitis [8]. Figure 1 describes the representation of acute pancreatitis and Balthazar grade scoring system. A normal -appearing pancreas (Grade A) was found in 33 patients (62%); Focal or diffuse enlargement of pancreas (Grade B) was found in 9 patients (17%); Abnormalities in pancreatic gland with peripancreatic inflammation (Grade C) was found in 5 patients (9%); Fluid collection in single location (Grade D) was found in 4 patients (7.5%); Multiple fluid collections was noticed in 2 patients (3.7%). Figure 2A and 2B are images from cuts of the CT scan of acute pancreatitis in a 41-year-old female showing lucencies in the head of the pancreas and peripancreatic fluid stranding.

## Discussion

New York City remains an epicenter of HIV/AIDS in the U.S. More than 107,000 New Yorkers are living with HIV, and thousands more don't know they're infected. New York City's AIDS case rate is almost three times that of the U.S. average, and HIV is the third leading cause of death for New York City for residents aged 35 to 54 [9]. Kings County (Brooklyn) is the second highest prevalence for HIV infection, with 37,183 reported AIDS cases [9]. Our center, the Treatment for Life Center (TLC) is a NY State Department of Health Designated AIDS Center, founded in 1993 serves 2000 HIV and AIDS patients every year.

We noticed total of 94 admissions in two years time period with HIV and AIDS presented with acute pancreatitis. Our findings are consistent with those previously a published by Reisler et al [7] and others group [5], that patients with immunosuppression as measured by CD4 < 50 cells/mm^3^, had a higher number of admissions with acute pancreatitis. This has significantly affected increased length of stay and morbidity compare to other groups. However, it is unclear what is the exact mechanism of increase pancreatitis in these patients.

Previous studies [5, 7] have reported female gender as a potential risk factor for acute pancreatitis but on the same note several others studies like Cappell and Marks [3] found an increased risk for pancreatitis in males. In our cohort we did not find any gender association with a Male: Female percentage ratio of 11:9.

History of alcohol intake was noticed in half of our patients and 20% had undergone cholecystectomy, both of which are also known risk factors for pancreatic inflammation in non-HIV associated pancreatitis. However, our data and sample size was small therefore no particular trend was observed. None of our patients had significant hypertriglycedemia (> 1,000 mg/dL).

Prior to HAART therapy, several reports have suggested NRTIs like Didanosine and Stavudine associated with acute pancreatitis [1, 5, 7]. During the last decade, several studies have questioned about the pancreatitis whether cause by HAART therapy. To date, no supportive evidence suggests pancreatitis with HAART therapy. In our study, only third of patients were on HAART therapy and only 18 patients on PCP prophylaxis, suggesting other risk factors are contributing at least equally to the development of acute pancreatitis in patients with a low CD4. Other opportunistic infections like Cytomegalovirus (CMV), Mycobacterium avium complex, tuberculosis, cryptosporidiosis, toxoplasmosis and cryptococcus have been linked to pancreatitis in various case reports [6].

In our study, one patient had cryptococcoal infection and one had *Mycobacterium avium*- complex infection presented with recurrent pancreatitis. One of the important observations in our study was the high prevalence of hepatitis C virus and Hepatitis B virus infections in our patients. Previously, Reisler et al [7] have reported the similar finding in their study cohort. The Increased prevalence of hepatitis B and C virus has raised several questions about the severity of acute pancreatitis in these patients. It is also possible that HIV virus by itself or concomitant infection with viral hepatitis B and hepatitis C can potentiate pancreatitis.

Autopsy studies in asymptomatic children have shown severe pancreatic lesions in a large number of these HIV and AIDS (61/73) HIV infected children without clinical evidence of pancreatic disorders [9-12]. Chehter et al [13] studied 109 patients with AIDS and 38 controls and found out acinar atrophy (60%), few zymogen granula in acinar cytoplasm (52%), abnormalities in acinar nucleus (65%), pancreatic steatosis (66%), and focal necrosis (17%). Immunohistochemistry revealed: mycobacteriosis (22%), toxoplasmosis (13%), cytomegalovirus (9%), Pneumocystis carinii (9%), and HIV p24 antigen in macropahge cytoplasm (22%). Ultrastructural examination showed: decreased zymogen granula, enlargement and proliferation of the endoplasmic reticulum and mitochondria, nuclear abnormalities, and increased lipid droplets in acinar cytoplasm. Necrosis, edema, and hemorrhage were not seen. Three morphologic patterns of pancreatic changes were identified: “nutritional-like,” inflammatory, and mixed (coexisting nutritional-like and inflammatory patterns). Surprisingly, authors did not find correlation between morphologic findings and clinical symptoms. Similar group later on perform ultrastructural study on pancreas for protein malnutrion and found decreased zymogen granules, acinar atrophy, increased lipofucsin pigment, and rarefying Golgi complex suggesting the morphologic substrate of protein-energy malnutrition in AIDS patients [14].

Future studies should study the correlation of pancreatitis with higher viral loads and correlate with patients with symptomatic pancreatitis to establish the etiology of association of HIV virus replication and pancreatic changes.

There are several potential limitations to our study. First, these results are based on patients from a single institution with a high proportion of patients belonging to a disadvantaged urban setting. Our sample size is relatively smaller for assessing the risk factors and association for pancreatitis; however provides useful information about the prevalence of the factors that can cause pancreatitis. The study is further limited to administrative data that the authors were unable to validate their diagnoses by confirming elevated levels of serum amylase or lipase or a corroborating clinical syndrome.

### Conclusion

Our study have shown Immunsuppression by HIV virus as measured by CD4 count < 200 is associated with increased number of admissions with pancreatitis and CD4 < 50 is further associated with a longer length of stay. Gender did not emerge as a significant risk factor however co-infection with HBV and HCV appear significant factor for pancreatitis. This was an interesting observation and future studies need to focus on disease prevalence and severity in patients with HBV and HCV concomitant or super infection so more light can be shed on the mechanism of pancreatitis. Future studies should also focus on the association between viral load and pancreatic changes which may allow us to understand further the presentation of pancreatitis in HIV infected patients.

## Presentation

The study was presented at New York American College of Physician Chapter Meeting, Rochester, New York 2011.
